# Real‐world data of lapatinib and treatment after lapatinib in patients with previously treated HER2‐positive metastatic breast cancer: A multicenter, retrospective study

**DOI:** 10.1002/cam4.2943

**Published:** 2020-02-28

**Authors:** Yizhao Xie, Rui Ge, Die Sang, Ting Luo, Wei Li, Xuening Ji, Peng Yuan, Biyun Wang

**Affiliations:** ^1^ Department of Medical Oncology Department of Oncology Fudan University Shanghai Cancer Center Shanghai Medical College Fudan University Shanghai China; ^2^ Huadong Hospital Affiliated to Fudan University Shanghai China; ^3^ Department of Oncology San Huan Cancer Hospital Beijing China; ^4^ Department of Head, Neck and Mammary Gland Oncology Cancer Center West China Hospital Sichuan University Sichuan China; ^5^ Department of Medical Oncology Jiangsu Province Hospital Nanjing China; ^6^ Department of Oncology Affiliated Zhongshan Hospital of Dalian University Dalian China; ^7^ National Cancer Center Tumor Hospital of the Chinese Academy of Medical Sciences Beijing China

**Keywords:** chemotherapy, clinical study, Her2, lapatinib, metastatic breast cancer

## Abstract

Lapatinib is widely used in the later lines treatment of HER2 positive metastatic breast cancer (MBC). EGF104900 study suggested that among patients who experienced progression on prior trastuzumab‐containing regimens, lapatinib plus trastuzumab had better effects than trastuzumab alone. However, no evidence was discovered in terms of lapatinib plus capetabine compared with lapatinib plus trastuzumab plus chemotherapy, as well as a treatment after progression on lapatinib. We evaluated the medical records retrospectively of all MBC patients with HER2 positive disease who progressed on prior trastuzumab‐containing regimens (advanced setting) and a taxane (any setting) and received lapatinib‐based treatment from 2015 to 2018 in five institutions in China. A total of 242 patients were available for analysis. Among them, 164 (68%) patients received lapatinib plus capetabine (LX) and 78 (32%) patients received lapatinib plus trastuzumab and one chemotherapy (HLC). The median progression‐free survival (PFS) of the HLC group was significantly superior to the LX group (8.8 months vs 5.0 months, *P* < .0000001). No significant difference in grade 3 or worse adverse events was observed in two groups (*P* = .57). A total of 175 patients were available for the analysis of the postlapatinib treatment. Continuation of lapatinib showed superior mPFS results compared to the non‐anti‐HER2 treatment (4 months vs 2 months, *P* = .01) and similar results compared to switch to other anti‐HER2 treatments (4 months vs 4 months, *P* = .88). In patients who had progressed on prior trastuzumab‐base therapy, HLC provided a new dual‐targeting treatment option for the later lines therapy of patients with HER2 positive MBC. Moreover, evidence of cross‐line use of lapatinib was provided.

## Graphical abstract

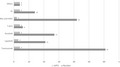

## 1 INTRODUCTION

Breast cancer (BC) is still the most common malignant tumor and a major cause of death among women worldwide, causing nearly 2.1 million new cases and 626 thousand deaths in 2018, mostly for metastatic breast cancer (MBC).^1^ In China, newly diagnosed breast cancer reached 278.9 thousand, and 66 thousand women died of it in 2014, according to the latest data.^2^

Amplification of the human epidermal growth factor receptor‐2 (HER2) expression occurs in 20%‐25% of primary breast cancers and is associated with poor clinical outcomes.^3^

Lapatinib is a small‐molecule, oral, reversible inhibitor targeting the adenosine triphosphate binding site within the intracellular kinase domain of ErbB1 (epidermal growth factor receptor) and ErbB2, which is widely used in the later lines of HER2‐positive MBC. In a randomized phase III EGF100151 trial, HER2‐positive MBC patients pretreated with trastuzumab‐based therapy were enrolled and the results showed that the time to progression (TTP) in lapatinib plus capecitabine (LX) group was significantly better than the capecitabine monotherapy group (95% confidence interval [CI], 0.43‐0.77; *P* < .01).^4^ This research proved the efficacy of LX among pretreated HER2 + MBC.

Preclinical studies explored the combination of trastuzumab and lapatinib. The two agents showed consistent strong synergistic interactions against HER‐2‐overexpressing established human breast cancer cell lines, with drug concentrations used between 0.039 and 5.0 Amol/L for lapatinib and 0.31 and 4.0 Ag/mL for trastuzumab.^5^ Another mechanism was found that Lapatinib‐induced accumulation of HER2 and trastuzumab‐mediated downregulation of HER2 triggered enhanced immune‐mediated trastuzumab‐dependent cytotoxicity in SKBR3 and MCF7‐HER2 cells.^6^

As for the clinical study, a phase III EGF104900 trial demonstrated that lapatinib plus trastuzumab (HL) had a statistically significant advantage over the group on lapatinib monotherapy in PFS (*P* = .008, 95% CI, 0.57‐0.93).^7^ This study indicated that the dual anti‐Her2 treatment of HL was superior to lapatinib alone. However, there was no comparison between LX compared with HL plus one chemo regimen (HLC).

Several studies indicated that the continuation of trastuzumab after progression on it had better efficacy compared with discontinuation and the reason might lie in the antibody‐dependent cell‐mediated cytotoxicity (ADCC) effect of trastuzumab.^8^, ^9^ Nevertheless, lapatinib had a different mechanism without ADCC. Will the continuation of lapatinib provide benefits for patients? There was no evidence of treatment after the progression on lapatinib yet.

Therefore, this study aims to figure out not only the efficacy and safety of LX and HLC among patients previously treated with trastuzumab and taxane but also the treatment pattern after the progression of lapatinib in clinical practice.

## 2 METHODS

### 2.1 Patients and treatments

We analyzed all patients treated with Lapatinib between 2015 and 2019 from databases at five institutions in China, including Fudan University Shanghai Cancer Center, Beijing San Huan Cancer Hospital, West China Hospital Sichuan University, Jiangsu Province Hospital and Affiliated Zhongshan Hospital of Dalian University. The eligibility criteria were as follows. (a) HER2 + MBC patients scored + 3 by immunohistochemical (IHC) analysis or scored + 2 and the result of fluorescence in situ hybridization was positive. (b) Patients were treated previously with a taxane in any setting and trastuzumab in the advanced setting. (c) Patients received lapatinib (1250 mg/day) plus capecitabine (2000 mg/m^2^) or lapatinib (750 mg‐1250 mg/day) plus intravenous trastuzumab 6 mg/kg (after the initial 8 mg/kg loading dose) per 21 days plus one chemo regimen (capecitabine, platinum, etc.), starting from 2015 to 2019. (d) Patients had complete medical records. Patients treated with two or more chemo regimens or with incomplete medical data were excluded. Medical data were retrospectively collected from the medical records systems of each institution.

### 2.2 Outcome measurements

Progression‐free survival (PFS) was the primary outcome measure of this study, defined as the time from initiation of Lapatinib‐based treatment to disease progression or death. The second outcome measure included overall survival (OS), safety, and treatment option as well as efficacy after the progression on Lapatinib. Overall survival (OS) was defined as the time between the initiation of treatment to death from any cause or censoring on 20 July 2019. Tumor evaluation was assessed by Response Evaluation Criteria in Solid Tumors (RECIST) 1.1 criteria. Adverse events (AEs) were analyzed according to the National Cancer Institute Common Terminology Criteria for Adverse Events (CTCAE) version 4.03. Disease‐free interval (DFI) was defined as the time from surgery to the diagnosis of metastasis. Trastuzumab resistance was defined as new recurrences diagnosed during or within 12 months after adjuvant trastuzumab or progression at first radiological reassessment or within 3 months after first‐line trastuzumab in the metastatic setting.^10^

### 2.3 Statistics

All patients meeting the criteria were evaluated. Therapy options in real‐world practice, as well as clinicopathologic characteristics, were summarized and compared between two groups by the Chi‐square test. PFS and OS were estimated using Kaplan–Meier curves and compared by the log‐rank test. Hazard ratios with two‐sided 95% confidence interval (CI) were calculated with unadjusted and adjusted Cox proportional hazards models. Cox regression modeling using stepwise selection was used for determining the effect of various baseline covariates on PFS and OS. Subgroup analysis was analyzed using the Cox regression model and showed by the forest plot. *P *< .05 was considered statistically significant. All statistical analyses were managed using SPSS version 23.0. Forest map was made by GraphPad Prism version 7.0.

## 3 RESULTS

### 3.1 Patients and treatment

The medical records of all patients using Lapatinib were retrospectively reviewed in five institutions and 242 patients were enrolled and evaluated in this study.

Among the 242 patients, 164 (68%) patients received LX and 78 (32%) patients received HLC. Patients and disease characteristics at the baseline between two treatment groups are shown in Table 1. The median age was 52 years for both groups. Most patients received surgery, while 15% of patients were de novo stage IV. Histology types were 85% of ductal carcinoma, 10% of lobular carcinoma, and 5% of others. Visceral metastases accounted for 72% and 73% of patients in the LX and HLC groups, respectively. Both groups received a median of two lines of prior treatment for MBC. Trastuzumab refractoriness was more common than trastuzumab resistance. Overall, no statistically significant differences were found in baseline characteristics between the two groups. No significant relation was found in the following factors considering the prescription preference of LX and HLC: Age ≥ or <60, DFI > or ≤2 years, ER status, visceral metastasis, and trastuzumab resistance.

**Table 1:** Baseline characteristics of patients grouped by LX or HLC

Characteristics	LX N = 164 n (%)	HLC N = 78 n (%)	*P* values
Median age (range)	52 (26‐86)	52 (28‐75)	.614
DFI			
<2 y	70 (43)	34 (44)	.936
≥2 y	70 (43)	32 (41)	
de novo stage IV breast cancer	24 (14)	12 (15)	
ECOG score			
0‐1	152 (92)	73 (94)	.622
≥2	14 (8)	5 (6)	
Visceral disease			
Yes	118 (72)	57 (73)	.855
No	46 (28)	21 (27)	
ER/PR status			
Positive	85 (52)	36 (46)	.462
Negative	79 (48)	42 (54)	
Median No. of prior treatment of metastatic disease (range)	2 (1‐10)	2 (1‐8)	.557
Trastuzumab resistance status			
Resistance	43 (26)	15 (19)	.170
Refractoriness	110 (67)	61 (78)	
Not known	11	2	

### 3.2 Treatment efficacy

**Figure 1:**
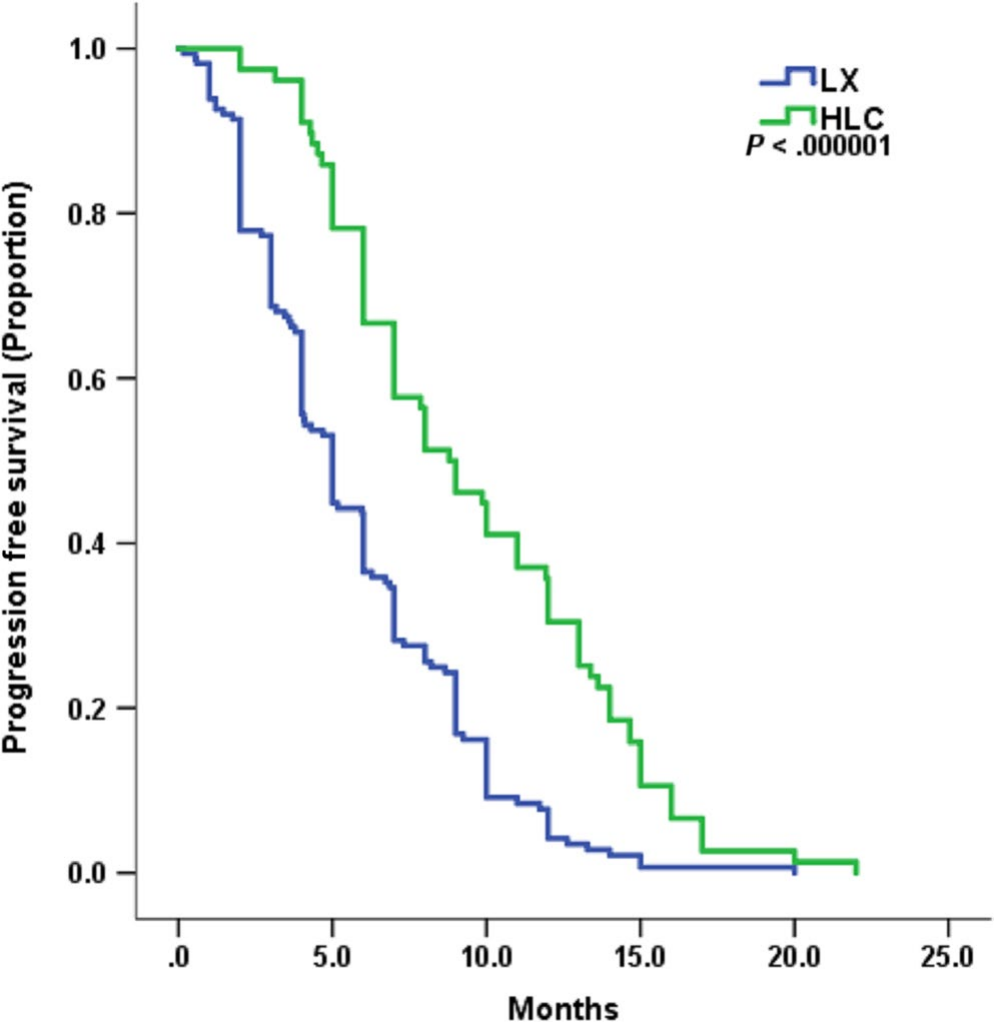
Kaplan–Meier curves for progression‐free survival by treatment arm of HLC and LX

At a median follow‐up period of 21 months, 156 of 164 patients in LX and 77 of 78 patients in HLC experienced progressive disease (PD). The combination of lapatinib with trastuzumab and one chemo regimen provided a statistically significant improvement in PFS compared with lapatinib and capecitabine, with an HR of 0.44 (95% CI, 0.33 to 0.59; *P* < .0000001; Figure 1). The median PFS was 5 months with LX compared with 8.8 months in HLC. The median OS was not reached at the time of analysis.

**Figure 2:**
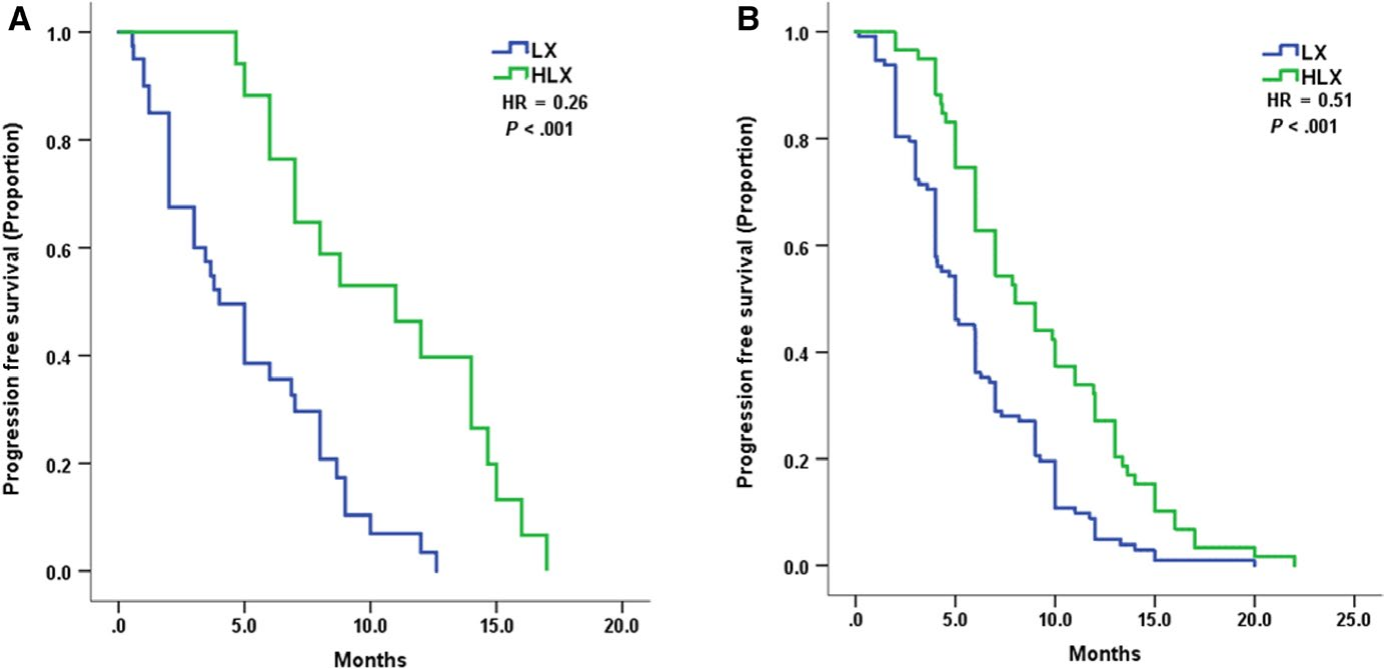
Kaplan–Meier curves for progression‐free survival by treatment arm of HLC and LX for patients with: A, trastuzumab resistance B, trastuzumab refractoriness

Trastuzumab resistance status was evaluated according to the treatment efficacy. In patients who had trastuzumab resistance, mPFS of LX and HLC was 4 months vs 11 months (*P* = .00032, HR = 0.26, 95%CI 0.12‐0.54). And in patients with trastuzumab refractoriness, mPFS of LX and HLC was 5 months vs 8 months (*P* = .000056, HR = 0.51, 95% CI 0.36‐0.71) (Figure 2).

**Figure 3:**
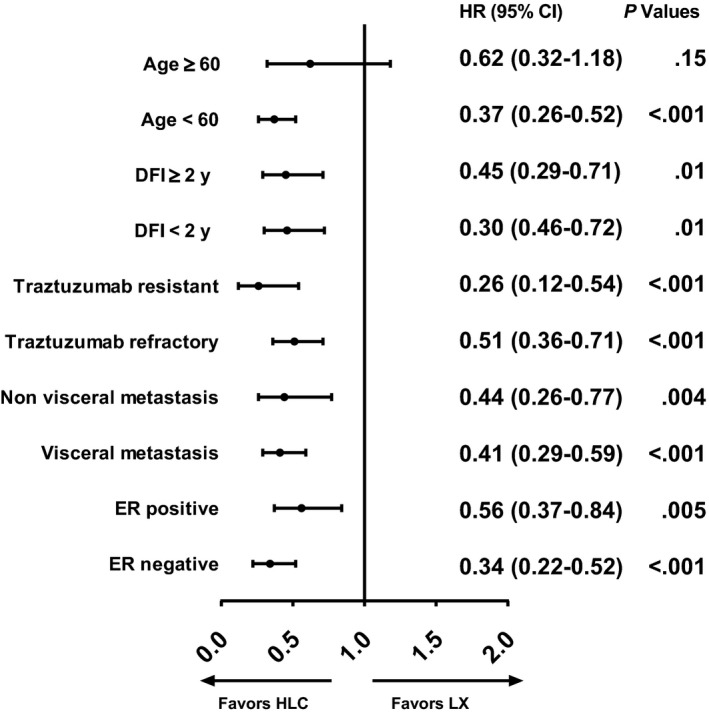
Forest plot of subgroup analysis including hazard ratios and 95% confidence intervals for PFS analysis

In subgroup analysis, the advantage of HLC over LX was maintained across most of the subsets. However, PFS of the LX group was similar to the HLC group among patients age ≥ 60 (mPFS 6 months vs 7 months, *P* = .11), compared to those age < 60 (mPFS 4.7 months vs 9 months, *P* < .0001). Furthermore, patients with ER + disease had fewer advantages of HLC over LX (mPFS 5.1 months vs 7 months, *P* = .005) than those with ER‐ disease (mPFS 5 months vs 10 months, *P* < .0001). The forest plot is shown in Figure 3.

In univariate analysis, HLC therapy (HR 0.44, 95% CI 0.33‐0.59 *P* < .0000001) and prior MBC treatment < 3 (HR 0.65, 95% CI 0.49‐0.89 *P* = .005) were predictive factors of longer PFS. In terms of multivariate analysis, HLC therapy (adjusted HR 0.42; 95% CI 0.31 to 0.57, *P* < .0000001) and prior MBC treatment < 3 (adjusted HR 0.60; 95% CI 0.44 to 0.81, *P* = .001) emerged as independent prognostic factors of lower risk of progression even after balancing the age, DFI, visceral metastasis, trastuzumab resistance, and ER status.

### 3.3 Safety

We evaluated the grade 3/4 adverse events (Table 2). Overall, both HLC and LX were well tolerated in our study, with only 16.5% of the LX group and 21.8% of the HLC group endured grade 3/4 AEs (*P* = .57). Common grade 3/4 side effects in LX patients were: diarrhea (5.5%), neutropenia (6.7%), leukopenia (6.1%), and palmar‐plantar erythrodysesthesia syndrome (3%) and in HLC patients were: neutropenia (11.5%), leukopenia (11.5%), diarrhea (7.7%), vomiting (3.8%), and leukopenia (6.4%).

**Table 2:** Adverse events (grade 3/4)

AE (grade 3/4)	LX N = 164 n (%)	HLC N = 78 n (%)
Diarrhea	9 (5.5)	6 (7.7)
Vomiting	2 (1.2)	3 (3.8)
Neutropenia	11 (6.7)	9 (11.5)
Leukopenia	10 (6.1)	9 (11.5)
Thrombocytopenia	1 (0.6)	0
Rash	1 (0.6)	1 (1.3)
Anemia	1 (0.6)	0
Palmar‐plantar erythrodysesthesia syndrome	5 (3.0)	1 (1.3)
Fatigue	1 (0.6)	0
Alanine aminotransferase increased	1 (0.6)	1 (1.3)
Dizziness	1 (0.6)	0
All	27 (16.5)	17 (21.8)

We used loperamide and octreotide to treat diarrhea, symptomatic treatment (recombinant human granulocyte colony‐stimulating factor, blood transfusion, etc.) for myelosuppression. Most of the adverse events were well controlled. No patients died of adverse events.

### 3.4 Treatment after progression

**Figure 4:**
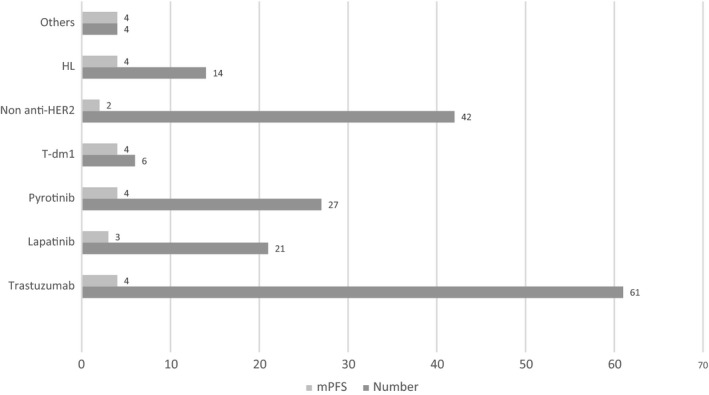
Treatment pattern of the postlapatinib setting including patient numbers and PFS (month) of each treatment

**Figure 5:**
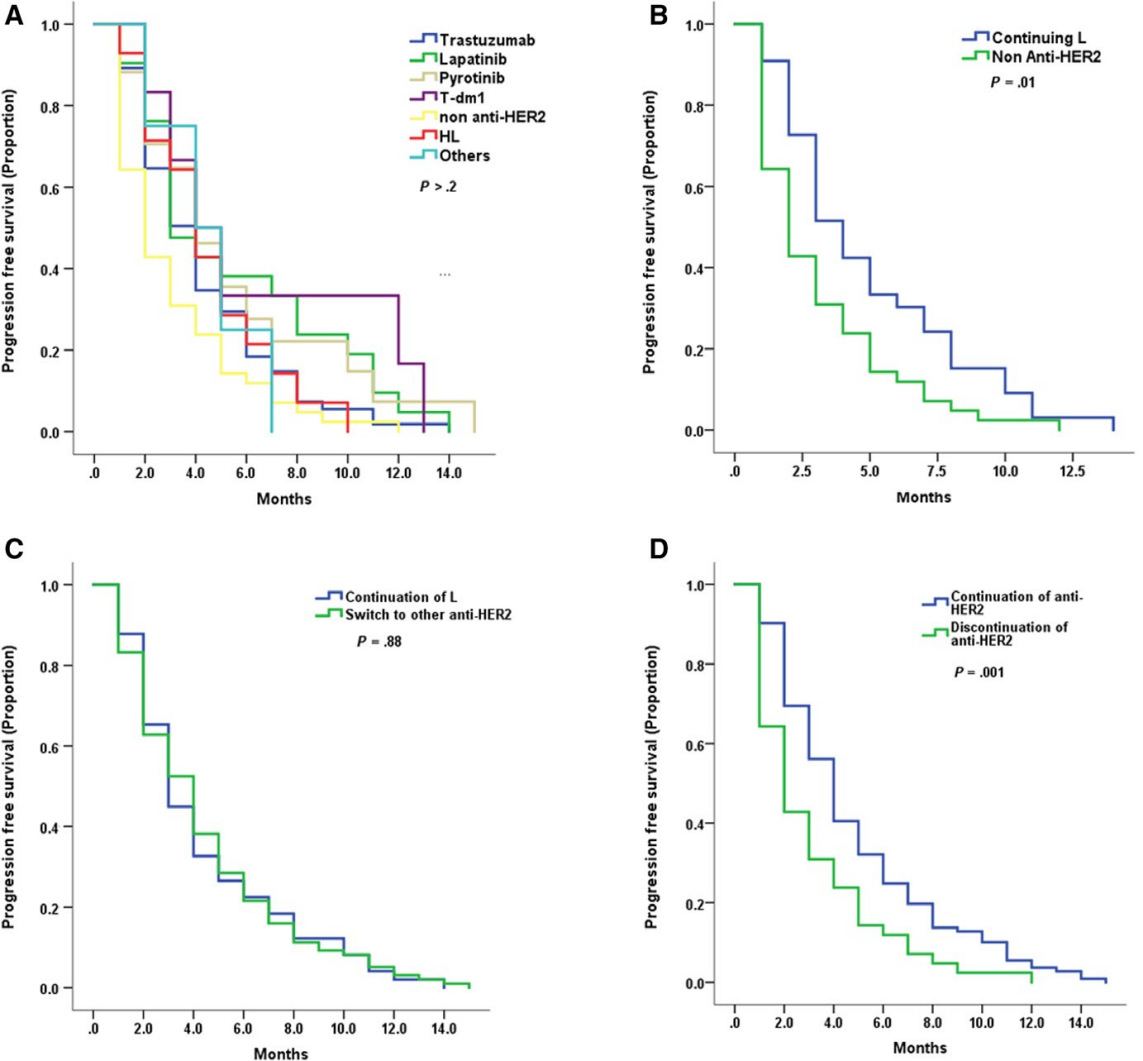
Kaplan–Meier curves for progression‐free survival by treatment arm for the postlapatinib setting patients with: A, All kinds of treatment (number of patients in Figure 4) B, Continuation of lapatinib (n = 35) or withdraw of anti HER2 treatment (n = 42) C, Continuation of lapatinib (n = 35) or change anti‐HER2 treatment (n = 98) D, Continuation (n = 133) or withdraw of anti HER2 treatment (n = 42)

We evaluated the treatment after the progression of lapatinib‐containing regimen. About 175 patients were available for analysis and 34.8% of patients received trastuzumab‐based regimen (n = 61), 19.4% of patients for pyrotinib‐based regimen (n = 27), 12% of patients received lapatinib (n = 21), 8% of patients received HL (n = 14), while 24% of patients did not continue anti‐HER2 treatment (n = 42). Detailed information and mPFS are shown in Figures 4 and 5A.

Continuation of lapatinib showed superior mPFS results compared to non‐anti‐HER2 treatment (4 months vs 2 months, HR 0.59, 95% CI 0.37‐0.94, *P* = .01, Figure 5B) and similar results compared to switch to other anti‐HER2 treatments (4 months vs 4 months, HR 0.97, 95% CI 0.70‐1.36, *P* = .88, Figure 5C). However, continuation of anti‐HER2 treatment had better PFS than discontinuation (4 months vs 2 months, HR 0.58, 95% CI 0.41‐0.83, *P* = .001, Figure 5D). Overall, anti‐HER2 treatment showed similar effects in terms of the postlapatinib setting.

## 4 DISCUSSION

Our study revealed the real‐world practice of lapatinib in combination with capecitabine or trastuzumab plus one chemotherapy and investigated the treatment after the lapatinib‐based regimen. As far as we are concerned, this is the first direct evaluation of comparison between LX and HLC, as well as the treatment after lapatinib for HER2 + MBC patients.

First and foremost, more patients (68%) received traditional LX treatment rather than the dual‐targeted HLC regimen (32%). The prescription of two treatments was not related to known baseline factors, which means doctors tend to use more LX than HLC in daily practice without a specific selection of patients. The reason could be less evidence and more costs of HLC. A perspective real‐world research in Germany showed that in 451 HER + MBC patients, 53% were treated with trastuzumab, 52% with pertuzumab and trastuzumab, 19% with lapatinib, and 28% with T‐DM1,^11^ which indicated more use of trastuzumab‐based therapy, mainly because most of the patients were in first‐line therapy. Real‐world data of later lines therapy for HER2 + MBC were not available. It is also noteworthy that T‐DM1 is not approved in China.

Our study revealed the superiority of HLC over LX in HER2 + MBC patients previously treated with trastuzumab and taxane regarding the significantly improved PFS. The previous study demonstrated the efficacy and safety of LX. The PFS results of randomized controlled trials (RCT) were consistent with our results. EGF100151 study enrolled 399 pretreated HER2 + MBC patients and the results showed that the mTTP was 6.0 months in the LX arm and 4.6 months in the X arm (*P* < .01), although no difference was found in OS.^4^ Based on this study, LX was recommended and widely used in clinical practice among pretreated HER2 + MBC patients. CEREBEL trial compared LX with trastuzumab plus capecitabine in HER2 + MBC patients and it indicated that in patients previously treated with trastuzumab, LX was similar to HX (6.6 months vs 6.1 months, HR 1.13, 95% CI 0.85‐1.5).^12^ However, this study did not reach the primary endpoint of CNS metastases and excluded patients with CNS metastases at baseline. In the EMILIA study, 991 pretreated HER2 + MBC patients were randomly assigned to the T‐DM1 group and LX group, and the results showed that the T‐DM1 group had better PFS than the LX group (9.6 months vs 6.4 months, *P* < .01), which roughly corresponded to our trial. However, in their study, some of the patients were treated in first‐line therapy for MBC, which could help explain the prolonged survival.^13^

The preclinical study found that treatment with HL might result in an enhanced anti‐tumor activity probably due to more complete blockage of HER2 signaling and synergistic drug interactions, for instance, lapatinib enhanced immune‐mediated trastuzumab‐dependent cytotoxicity, which could accumulate HER2 at the cell surface.^5^, ^6^ Despite the fact that no direct clinical evidence of HLC was reported, the combination of HL was explored in several trials. A phase III EGF104900 trial enrolled 296 heavily pretreated HER + MBC patients and showed that HL had a statistically significant advantage over lapatinib monotherapy in PFS (3 months vs 2 months, *P* = .008) but not in OS (12.9 months vs 9.7 months, *P* = .11).^7^ This study had a relatively low PFS mainly because patients had a median of four lines of previous treatment and no chemotherapy was added in the anti‐HER2 therapy. ALTERNATIVE study randomly assigned 355 HER2 + HR+MBC patients into HL plus aromatase inhibitor (AI), H plus AI and L plus AI.^14^ The results indicated that HL + AI was superior to H + AI (mPFS 11 vs 5.7 months, *P* = .0064). This trial figured out the efficacy and safety of dual‐anti HER2 treatment combined with endocrine therapy. The HLC group of our study had a similar PFS outcome, which is reasonable owing to the addition of chemotherapy.

Subgroup analysis suggested that the advantage of HLC maintained in both trastuzumab‐resistant group and trastuzumab‐refractory group confirmed the value of HLC regardless of the sensitivity of previous trastuzumab treatment. Given the fact that lapatinib had a synergistic effect when combined with trastuzumab, HLC might to some extent reverse the resistance of trastuzumab.

Less than 3 lines of prior MBC treatment was found to be a predictor of longer PFS in multivariate analysis. This finding conformed with previous trials considering prior lines of treatment that affect the treatment efficacy of lapatinib‐based regimen.^4^, ^7^

With regard to toxicity, both agents were well tolerated. Grade 3/4 AEs occurred in 16.5% of patients for LX and 21.8% of patients for HLC. The LX group had higher incidence of palmar‐plantar erythrodysesthesia syndrome, which is the common side effects of lapatinib when used with capecitabine. HLC had higher rates of gastrointestinal reaction, which was also seen in HL therapy.^7^ No grade 3/4 cardiac toxicity was observed in both groups, which indicated the cardiac safety of the HLC regimen.

Last but not least, we figured out the treatment pattern of the postlapatinib setting. Patients received trastuzumab, pyrotinib, and lapatinib most after the progression on lapatinib‐based treatment. Pyrotinib is a newly developed irreversible pan‐ErbB inhibitor, showing efficacy in HER2 + MBC with a mPFS of 18.1 months, which is widely used in China.^15^, ^16^ We found that the efficacy of the postlapatinib treatment was similar among different regimens, probably attributed to later lines, less sensitive to anti‐HER2 treatment, and limited sample size. Because of drug donation projects (giving free drugs after certain time of use), some patients might choose to use lapatinib even after progression. Moreover, patients continued using lapatinib had superior results compared to the non‐anti‐HER2 treatment group and similar results compared to other anti‐HER2 treatments. Our study suggested the eligibility for cross‐line use of lapatinib. Although lapatinib had no ADCC effect, sustained blockage of dual kinase inhibitor of both EGFR and HER‐2 might help. Furthermore, this study revealed that the continuation of anti‐HER2 treatment was superior to quitting it, which confirmed the necessity of sustaining blockage of HER2 signaling during the whole course of treatment among HER2 + MBC patients.

Since this study is retrospective, randomized controlled trials are warranted to provide more evidence. Moreover, drugs not available in China might result in the distinction of treatment pattern compared to other regions. Additionally, more emphasis is warranted for optimal sequences and new targeted regimens for the sake of a better life span of breast cancer patients.

In conclusion, this study revealed the real‐world practice of LX and HLC among pretreated HER2 + MBC patients, as well as the treatment after lapatinib. In patients who had progressed on prior trastuzumab‐base therapy, HLC resulted in a significant improvement in PFS vs LX with tolerable adverse events, thus offering a new dual‐targeting treatment option for later lines therapy of patients with HER2 positive MBC. No significant difference was found in kinds of therapies after lapatinib and patients continued using lapatinib had superior results compared to the non‐anti‐HER2 treatment group and similar effect compared with other anti‐HER2 treatments. However, the continuation of anti‐HER2 treatment was proved better than quitting it. Our study could provide precious evidence for doctors in terms of the medical decision in the future.

## CONFLICT OF INTEREST

The authors declare no competing financial interests.

## AUTHOR CONTRIBUTIONS

Yizhao Xie collected all of the data from different centers, performed the statistical analysis, and finished the manuscript. Rui Ge participated in the manuscript and checked the English grammar in the manuscript. Die Sang, Ting Luo, Wei Li, and Xuening provided the data and involved in data analysis. Biyun Wang and Peng Yuan designed, carried out the study and revised the manuscript. This research is registered at clinicaltrials.gov (NCT 03894410).

## DECLARATIONS

Ethics Committee and Institutional Review Boards in the Fudan University Shanghai Cancer Center approved this study for clinical investigation. Ethics approval and consent to participate all procedures performed in studies involving human participants were in accordance with the ethical standards of the institutional research committee and with the 1964 Helsinki declaration and its later amendments or comparable ethical standards. The need for written informed consent was waived as it is a retrospective study.

## Data Availability

The datasets generated and analyzed during the current study are not publicly available due to hospital policy but are available from the corresponding author on reasonable request.

## References

[1] Bray F , Ferlay J , Soerjomataram I , Siegel RL , Torre LA , Jemal A . Global cancer statistics 2018: GLOBOCAN estimates of incidence and mortality worldwide for 36 cancers in 185 countries. CA Cancer J Clin. 2018;68(6):394‐424. 30207593 10.3322/caac.21492

[2] Chen W , et al. Cancer statistics in China, 2015. CA Cancer J Clin, 2016 66(2):115‐132. 26808342 10.3322/caac.21338

[3] Owens MA , Horten BC , Da SM . HER2 amplification ratios by fluorescence in situ hybridization and correlation with immunohistochemistry in a cohort of 6556 breast cancer tissues. Clin Breast Cancer. 2004;5(1):63‐69. 15140287 10.3816/cbc.2004.n.011

[4] Cameron D , Casey M , Oliva C , Newstat B , Imwalle B , Geyer CE . Lapatinib plus capecitabine in women with HER‐2‐positive advanced breast cancer: final survival analysis of a phase III randomized trial. Oncologist. 2010;15(9):924‐934. 20736298 10.1634/theoncologist.2009-0181 PMC3228041

[5] Konecny GE , Pegram MD , Venkatesan N , et al. Activity of the dual kinase inhibitor lapatinib (GW572016) against HER‐2‐overexpressing and trastuzumab‐treated breast cancer cells. Can Res. 2006;66(3):1630‐1639. 10.1158/0008-5472.CAN-05-1182 16452222

[6] Scaltriti M , Verma C , Guzman M , et al. a HER2 tyrosine kinase inhibitor, induces stabilization and accumulation of HER2 and potentiates trastuzumab‐dependent cell cytotoxicity. Oncogene. 2009;28(6):803‐814. 19060928 10.1038/onc.2008.432

[7] Blackwell KL , Burstein HJ , Storniolo AM , et al. Randomized study of lapatinib alone or in combination with trastuzumab in women with ErbB2‐positive, trastuzumab‐refractory metastatic breast cancer. J Clin Oncol. 2010;28(7):1124‐1130. 20124187 10.1200/JCO.2008.21.4437

[8] Extra J‐M , Antoine EC , Vincent‐Salomon A , et al. Efficacy of trastuzumab in routine clinical practice and after progression for metastatic breast cancer patients: the observational hermine study. Oncologist. 2010;15(8):799‐809. 20671105 10.1634/theoncologist.2009-0029 PMC3228018

[9] von Minckwitz G , du Bois A , Schmidt M , et al. Trastuzumab beyond progression in human epidermal growth factor receptor 2‐positive advanced breast cancer: a german breast group 26/breast international group 03–05 study. J Clin Oncol. 2009;27(12):1999‐2006. 19289619 10.1200/JCO.2008.19.6618

[10] Wong H , et al. Integrating molecular mechanisms and clinical evidence in the management of trastuzumab resistant or refractory HER‐2(+) metastatic breast cancer. Oncologist. 2011;16(11):1535‐1546. 22020213 10.1634/theoncologist.2011-0165 PMC3233287

[11] Lux M , Nabieva N , Hartkopf A , et al. Therapy landscape in patients with metastatic HER2‐positive breast cancer: data from the PRAEGNANT real‐world breast cancer registry. Cancers. 2019;11(1):10. 10.3390/cancers11010010 PMC6357172 30577662

[12] Pivot X , et al. CEREBEL (EGF111438): a phase III, randomized, open‐label study of lapatinib plus capecitabine versus trastuzumab plus capecitabine in patients with human epidermal growth factor receptor 2‐positive metastatic breast cancer. J Clin Oncol. 2015;33(14):1564‐1573. 25605838 10.1200/JCO.2014.57.1794

[13] Verma S , Miles D , Gianni L , et al. Trastuzumab emtansine for HER2‐positive advanced breast cancer. N Engl J Med. 2012;367(19):1783‐1791. 23020162 10.1056/NEJMoa1209124 PMC5125250

[14] Johnston SRD , Hegg R , Im S‐A , et al. Phase III, randomized study of dual human epidermal growth factor receptor 2 (HER2) blockade with lapatinib plus trastuzumab in combination with an aromatase inhibitor in postmenopausal women with HER2‐positive, hormone receptor‐positive metastatic breast cancer: Alternative. J Clin Oncol. 2018;36(8):741‐748. 29244528 10.1200/JCO.2017.74.7824 PMC7444639

[15] Binghe Xu EA . Phase II clinical trial of pyrotinib, in San Antonio Breast Cancer Symposium, PD 3–08. 2017.

[16] Li Q , Guan X , Chen S , et al. Safety, efficacy, and biomarker analysis of pyrotinib in combination with capecitabine in HER2‐positive metastatic breast cancer patients: a phase I clinical trial. Clin Cancer Res. 2019;25(17):5212‐5220. 31138588 10.1158/1078-0432.CCR-18-4173

